# Ascorbic Acid Ameliorates Molecular and Developmental Defects in Human-Induced Pluripotent Stem Cell and Cerebral Organoid Models of Fragile X Syndrome

**DOI:** 10.3390/ijms252312718

**Published:** 2024-11-26

**Authors:** Keith M. Gunapala, Aseel Gadban, Faiza Noreen, Primo Schär, Nissim Benvenisty, Verdon Taylor

**Affiliations:** 1 Department of Biomedicine, University of Basel, Mattenstrasse 28, 4058 Basel, Switzerland; keith.gunapala@unibas.ch (K.M.G.); faiza.noreen@unibas.ch (F.N.); primo.schaer@unibas.ch (P.S.); 2 The Azrieli Center for Stem Cells and Genetic Research, Department of Genetics, Institute of Life Sciences, The Hebrew University, Jerusalem 91904, Israel; aseel.gadban@mail.huji.ac.il; 3 Swiss Institute of Bioinformatics, 4031 Basel, Switzerland

**Keywords:** FMR1, Fragile X Syndrome, methylation, Ascorbic Acid, gene silencing, Autism Spectrum Disorders (ASD), induced pluripotent stem cells, cerebral organoids, neurodevelopmental disorders

## Abstract

Fragile X Syndrome (FX) is the most common form of inherited cognitive impairment and falls under the broader category of Autism Spectrum Disorders (ASD). FX is caused by a CGG trinucleotide repeat expansion in the non-coding region of the X-linked *Fragile X Messenger Ribonucleoprotein 1* (*FMR1*) gene, leading to its hypermethylation and epigenetic silencing. Animal models of FX rely on the deletion of the *Fmr1* gene, which fails to replicate the epigenetic silencing mechanism of the *FMR1* gene observed in human patients. Human stem cells carrying FX repeat expansions have provided a better understanding of the basis of epigenetic silencing of *FMR1*. Previous studies have found that 5-Azacytidine (5Azac) can reverse this methylation; however, 5Azac can be toxic, which may limit its therapeutic potential. Here, we show that the dietary factor Ascorbic Acid (AsA) can reduce DNA methylation in the *FMR1* locus and lead to an increase in *FMR1* gene expression in FX iPSCs and cerebral organoids. In addition, AsA treatment rescued neuronal gene expression and morphological defects observed in FX iPSC-derived cerebral organoids. Hence, we demonstrate that the dietary co-factor AsA can partially revert the molecular and morphological defects seen in human FX models in vitro. Our findings have implications for the development of novel therapies for FX in the future.

## 1. Introduction

Fragile X Syndrome (FX) is the most common form of genetically inherited cognitive impairment and mental retardation, with a prevalence of 1 in 3600 in the population (varying by geographic region), and is one of the major forms of Autism Spectrum Disorders (ASD) [1]. FX patients display a constellation of symptoms and phenotypes, including severe intellectual disability, attention deficits, problems with speech and social interaction, aggression, hyperactivity, and high susceptibility to seizures [2,3]. FX has a massive impact on the lives of both patients and their families.

**Figure 1:**
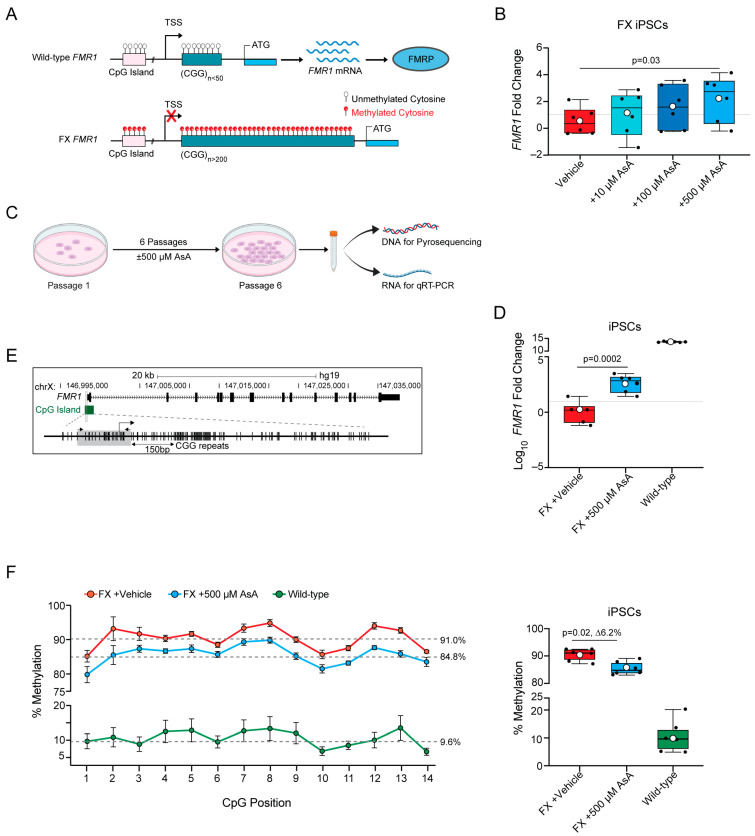
Ascorbic Acid restores *FMR1* expression by reversing hypermethylation in FX iPSCs. (**A**) Schematic representation the gene structure of human *FMR1* in wild-type and FX. In wild-type *FMR1* (top section of the panel), there are less than 50 CGG repeats and no methylation of the CGG repeats and CpGs upstream; therefore, the gene is transcriptionally active, producing *FMR1* mRNA and the FMRP protein product. In FX *FMR1* (lower section of the panel), there are >200 CGG repeats that are hypermethylated; hypermethylation spreads upstream to the CpGs, and therefore, the gene is transcriptionally inactive. Unmethylated Cytosine is represented by transparent circular marks over the CpG and CGG sequence; methylated Cytosine is represented by red circular marks over the CpG and CGG sequence. (**B**) Dose−response curve for FX iPSCs treated for 1 passage (6 days) with Ascorbic Acid (AsA). FX vehicle control with 15 mM HEPES (red), 10 μM AsA (n = 6, turquoise), 100 μM AsA (n = 6, navy blue), or 500 μM AsA (n = 6, blue). Values plotted are fold changes of *FMR1* expression normalized to the FX vehicle control. (**C**) Schematic representation the timeline and experimental setup of long-term exposure to AsA. Low-density iPSCs are plated and kept in culture for 6 days and passaged, repeating until the sixth passage when cells are then collected for either DNA extraction (to perform pyrosequencing) or RNA extraction (to perform qRT-PCR). (**D**) *FMR1* gene expression fold change (in log_10_) from FX vehicle control (n = 6, red) and FX + 500 μM AsA (n = 6, blue), and wild-type (n = 6, green). (**E**) Schematic of the *FMR1* gene structure with upstream CGG repeats and CpG island (in green) 150 base pairs upstream of the CGG. CpG island in green is used as a proxy to quantify methylation of the *FMR1* locus. The gray box highlights the region of the CpG that was sequenced to quantify methylation levels by pyrosequencing. The arrows (black) show the locations of the forward and reverse primers. (**F**) Left panel: average methylation levels per CpG site across all samples of iPSCs. FX vehicle-control (n = 6, red); FX + 500 μM AsA (n = 6, blue) and wild-type (n = 6, green). Data are presented for 14 CpG sites, with error bars indicating the standard error of the mean (±SEM). Gray dashed lines represent the median percentage methylation across all CpGs for each group. Right panel, boxplot showing the percentage methylation level for each individual sample: FX vehicle control (n = 6, red), FX + 500 μM AsA (n = 6, blue), and wild-type (n = 6, green). All boxplots display the interquartile range (IQR) and median, with the mean indicated by white circles. *P*-values were calculated using the Wilcox rank-sum exact test.

Unlike most forms of ASD, FX has a well-established monogenic cause. A CGG trinucleotide repeat expansion in the 5′ untranslated region (UTR) of the *Fragile X Messenger Ribonucleoprotein 1* (*FMR1)* gene leads to its epigenetic silencing and consequently results in FX [4,5]. However, *FMR1* gene expression shows a CGG repeat expansion dosage effect. The normal *FMR1* gene contains 5–50 CGG repeats. An expansion to 55–200 repeats is referred to as the pre-mutation state, resulting in a reduction in Fragile X Mental Retardation Protein (FMRP) levels and mild cognitive defects, but not FX [6,7,8]. Expansion beyond 200 CGG repeats induces DNA hypermethylation and epigenetic changes in the *FMR1* promoter, silencing the gene and causing FX (Figure 1A) [4,5,9]. DNA hypermethylation spreads to the entire locus, causing chromatin condensation and making the *FMR1* gene and locus inaccessible to the transcriptional machinery [9]. This genetic causation in FX presents advantages for identifying genes involved in autism-like disorders and devising therapeutic approaches. Since FX is caused by epigenetic silencing of *FMR1* and not by a mutation in the coding region, a therapeutic strategy could be to reverse hypermethylation of the gene locus and thereby reactivate the silenced gene. Mouse models of FX have partial or complete deletions of the *Fmr1* gene to prevent the expression of functional FMRP [10,11,12,13]. Hence, these models are not suitable for studying the mechanisms controlling hypermethylation or epigenetic silencing of the *FMR1* gene in humans. Human embryonic stem cells (ESCs) or induced pluripotent stem cells (iPSCs) that carry >200 CGG repeat expansions are powerful models for studying FX [14,15]. This is supported by the finding that FX ESCs and iPSC-derived neurons exhibit epigenetic silencing of *FMR1* [14,15]. Screening of FX iPSCs and their neuronal derivatives has identified several candidate compounds that can reactivate *FMR1* gene expression [16,17,18]. The cytosine analog and methyltransferase inhibitor 5-AzaC increased *FMR1* expression from 0% to 15–45% of the control levels in FX cells [16]. Genetic approaches, such as CRISPR-editing to excise the expanded CGG repeat region in the FX *FMR1* gene [19] and dCas9 to guide demethylases to the locus, have also demonstrated success in reactivating *FMR1* expression [20]. These studies highlight that demethylation of the hypermethylated *FMR1* gene in FX cells can restore its activity [16,17,18,19,20].

In previous studies, Ascorbic Acid (AsA) has been shown to affect chromatin conformation/reprogramming [21,22,23,24,25] or DNA methylation [26,27]. We hypothesized that AsA may be able to demethylate the *FMR1* locus in FX cells. In this study, we treated FX iPSCs and cerebral organoids with AsA and evaluated its effects on *FMR1* methylation status, mRNA expression, and organoid morphology. We found that AsA reduces *FMR1* methylation and increases *FMR1* expression in FX iPSCs, which has a similar effect in FX cerebral organoids. Furthermore, AsA treatment upregulated genes previously identified to be downregulated in FX and partially rescued the morphological development of FX cerebral organoids.

## 2. Results

### 2.1. Ascorbic Acid Restores FMR1 Expression by Reversing Hypermethylation in FX iPSCs

The *FMR1* gene contains 5–50 CGG repeats in the 5′UTR of the gene and an upstream CpG island in the gene promoter (Figure 1A). Expansion of CGG triplicate repeats to >200 in the *FMR1* gene leads to hypermethylation of the CGG repeats, which spreads to the upstream CpG island and the entire locus, resulting in transcriptional silencing and loss of the FMRP protein [4,5,9] (Figure 1A). To investigate whether Ascorbic Acid (AsA) can reverse the hypermethylation and transcriptional silencing of the *FMR1* gene in FX iPSCs, we treated FX iPSCs with different concentrations of AsA and quantified *FMR1* mRNA levels by quantitative RT-PCR.

AsA was administered to FX iPSCs daily for 6 days. To prevent acidification of the medium by AsA, HEPES was added to the culture medium, serving as a vehicle control. AsA induced *FMR1* transcription in a dose-dependent manner, with the highest level of *FMR1* mRNA expression observed at 500 μM AsA (Figure 1B). We speculated that prolonged treatment of iPSCs with AsA could further increase the activation of the *FMR1* gene in FX iPSCs. To test this, we plated FX and wild-type iPSCs in the presence or absence of 500 μM AsA and cultured them for 6 days until confluency. We expanded the cells for six passages in the presence or absence of AsA and isolated genomic DNA for pyrosequencing to analyze the methylation status and RNA to determine *FMR1* gene expression by quantitative RT-PCR (Figure 1C). Long-term AsA treatment over six passages resulted in a statistically significant increase in *FMR1* transcript levels compared to FX iPSCs treated with the vehicle control (Figure 1D).

We next examined whether long-term exposure of FX iPSCs to AsA could lead to demethylation of the *FMR1* gene locus. Due to the repetitive nature and length of the CGG repeats in *FMR1*, the CpG island upstream of the CGG repeats was used in pyrosequencing as a proxy to assess the methylation status of the *FMR1* gene (Figure 1E) [20]. The CpG island in wild-type iPSCs was unmethylated but hypermethylated in FX iPSCs (Figure 1F). AsA treatment of FX significantly reduced DNA methylation of the CpG island in FX iPSCs, suggesting that AsA has the potential to restore *FMR1* gene expression in FX iPSCs by reducing DNA methylation (Figure 1F).

### 2.2. Ascorbic Acid Reactivates FMR1 and Reduces Methylation in FX Cerebral Organoids

**Figure 2:**
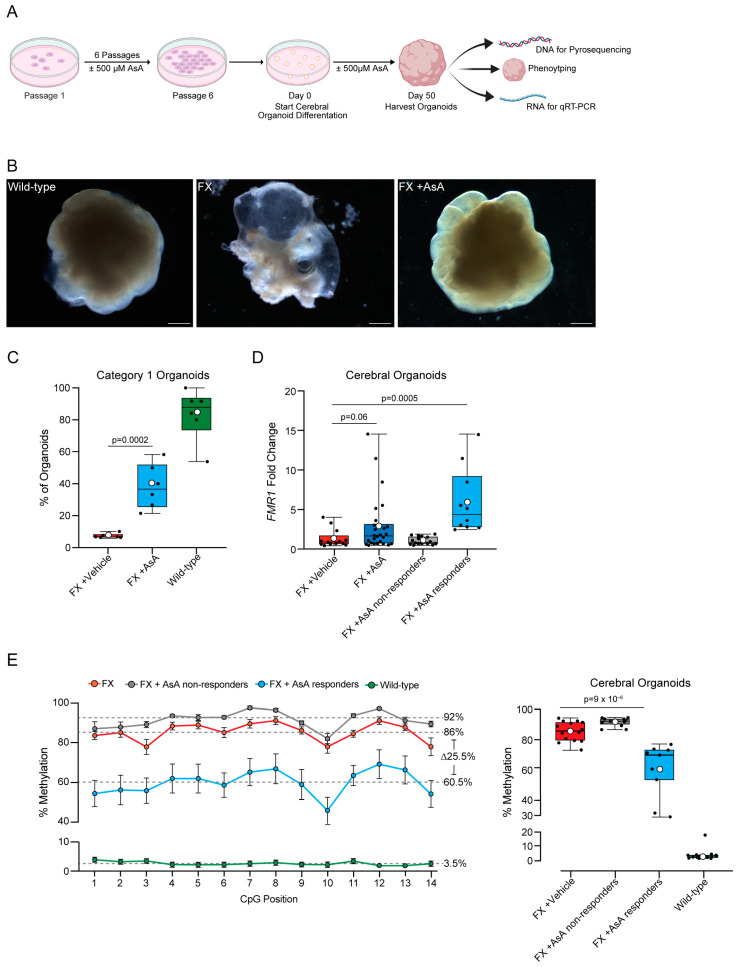
Ascorbic Acid reactivates *FMR1* and reduces methylation in FX cerebral organoids. (**A**) Schematic representation of the timeline and experimental setup for long-term exposure to AsA. Low-density iPSCs were plated and kept in culture for 6 days, passaged, and repeated until the sixth passage, where cells were then used to start the cerebral organoid differentiation protocol. Cerebral organoids are cultured until day 50, and they are collected for either DNA extraction (to perform pyrosequencing) or RNA extraction (to perform qRT-PCR) or imaged for phenotype assessment. (**B**) Representative images of wild-type (left panel), FX vehicle control (center panel), and FX + AsA (right panel) cerebral organoids. Scale bar for wild-type: 2200 μm, 3000 μm for FX, and 3000 μm for FX + AsA. (**C**) Percent of cerebral organoids per plate that had morphologically organized neuronal tissue by visual scoring of phenotype; FX vehicle control (n = 6, red), FX + 500 μM AsA (n = 6, blue), and wild-type (n = 6, green). n = 6 plates per genotype and condition; each plate contained 10–20 cerebral organoids. (**D**) *FMR1* gene expression fold change between FX vehicle control cerebral organoids (n = 13, red), all FX + 500 μM AsA-treated cerebral organoids (n = 26, navy blue), and FX + 500 μM AsA-treated cerebral organoids stratified by the 95% CI of FX vehicle controls into FX + AsA non-responders (n = 16, gray) and FX + AsA responders (n = 10, blue). (**E**) Left panel: average percentage of methylation levels at each CpG site across all samples for FX (n = 14, red), non-responders (n = 11, gray), responders (n = 9, blue), and wild-type (n = 14, green). Data are presented for 14 CpG sites, with error bars indicating the standard error of the mean (± SEM). Gray dashed lines represent the median percentage methylation across all CpGs for each group. On the right panel is a boxplot showing the percentage methylation level for each sample. Individual samples are represented by black circles. The boxplot displays the interquartile range (IQR) and median, with the mean indicated by white circles. *P*-values were calculated using the Wilcoxon rank-sum exact test.

Given that FX is a neurodevelopmental disorder, we used a relevant human model to assess the effects of AsA treatment on *FMR1* in cerebral organoids. Cerebral organoids have been shown to be an effective system for modeling human neurodevelopmental disorders [28,29,30]. We differentiated FX iPSCs that had been chronically treated with ±AsA for six passages into cerebral organoids. The cerebral organoids were exposed to ±500 μM AsA throughout their differentiation and collected on day 50 for analysis (Figure 2A).

We assessed the formation and morphology of cerebral organoids derived from wild-type, FX vehicle-treated, and AsA-treated FX iPSCs on day 50 of differentiation (Figure 2A). We classified the organoids into two categories: (category 1) cerebral organoids with smooth contours and distinct visible neural structures, and (category 2) cerebral organoids that are disorganized, mostly cystic, and lacking visible neural structures (Supplementary Figure S1A). These criteria were based on previously established phenotypic scoring using bright-field microscopy [28,29,30,31,32,33,34].

The majority of cerebral organoids derived from wild-type iPSCs (83.6 ± 6.6%) showed organized structures with smooth, well-defined borders (Figure 2B) and were classified as category 1 (Figure 2C). In contrast, only a small fraction of FX vehicle-treated cerebral organoids (7.3 ± 0.6%) were classified as category 1, with most appearing highly disorganized and cystic (Figure 2B,C). Notably, AsA-treated FX iPSC-derived cerebral organoids showed a marked improvement in phenotype with defined morphology (38.3 ± 5.7%) (Figure 2B), had defined organization and smooth boundaries, and were classified as category 1 (Figure 2C). However, the phenotypic rescue of FX cerebral organoids by AsA treatment was highly variable.

In order to assess the effects of AsA treatment on *FMR1* gene expression, we quantified *FMR1* mRNA levels in 50-day-old cerebral organoids. An initial comparison of *FMR1* transcript levels between FX vehicle- and AsA-treated cerebral organoids indicated a trend toward increased *FMR1* expression in response to AsA treatment (Figure 2D). However, the effects of AsA treatment were variable across individual organoids. Therefore, we used a threshold of the 95% confidence interval (CI) of the median *FMR1* expression in vehicle-treated FX cerebral organoids to define a threshold and identify AsA-treated cerebral organoids with increased *FMR1* expression. 38% of AsA-treated FX cerebral organoids exceeded the threshold of 95% CI (Figure 2D). These cerebral organoids were considered AsA responders. However, we identified two distinct populations of FX cerebral organoids: AsA responders and non-responders.

Next, we analyzed the methylation status of the CpG island upstream of the *FMR1* gene in cerebral organoids derived from AsA-treated FX, vehicle-treated FX, and wild-type iPSC using pyrosequencing. In wild-type cerebral organoids, the CpG island showed a low average methylation level (3.5 ± 1.3%) (Figure 2E). In contrast, the CpG island in FX cerebral organoids was highly methylated (86 ± 1.7%) (Figure 2E, Supplementary Figure S1B).

Wild-type cerebral organoids displayed a homogeneous and low methylation range (1.8–4.4%), except for one organoid, with a higher average methylation of 17.1% (Figure 2E, Supplementary Figure S1B). FX vehicle-treated cerebral organoids exhibited a uniformly high level of methylation in the CpG island (73.2–94.1%, average 86 ± 1.7%, Figure 2E, Supplementary Figure S1B).

AsA-treated cerebral organoids displayed a broader range of *FMR1* CpG island methylation (29.0–94.6%) with an average trend toward decreased methylation (Figure 2E, Supplementary Figure S1C). As AsA treatment of FX cerebral organoids resulted in heterogeneity within the population, we again used the 95% CI of vehicle-treated FX cerebral organoids to set a threshold and separate putative AsA responders from non-responders based on methylation status. 45% of AsA-treated FX cerebral organoids were classified as responders, with methylation levels similar to those of wild-type cerebral organoids (Figure 2E, Supplementary Figure S1C). Four AsA-treated cerebral organoids had CpG island methylation levels very similar to those of wild-type organoids (29.0, 31.5, 53.6, and 60.6% residue methylation over the entire island). In conclusion, combining the *FMR1* expression and methylation data confirms that cerebral organoids derived from FX iPSCs respond differentially to AsA treatment, resulting in two populations: responders and non-responders.

### 2.3. Ascorbic Acid Treatment Modulates Gene Expression in FX Cerebral Organoids Related to Neurodevelopment

In order to characterize the changes induced by AsA treatment in FX cerebral organoids, we performed RNASeq on individual AsA responders identified by their higher *FMR1* expression levels. We compared the gene expression profiles of these AsA-treated FX cerebral organoids to those of vehicle-treated FX and wild-type cerebral organoids.

**Figure 3:**
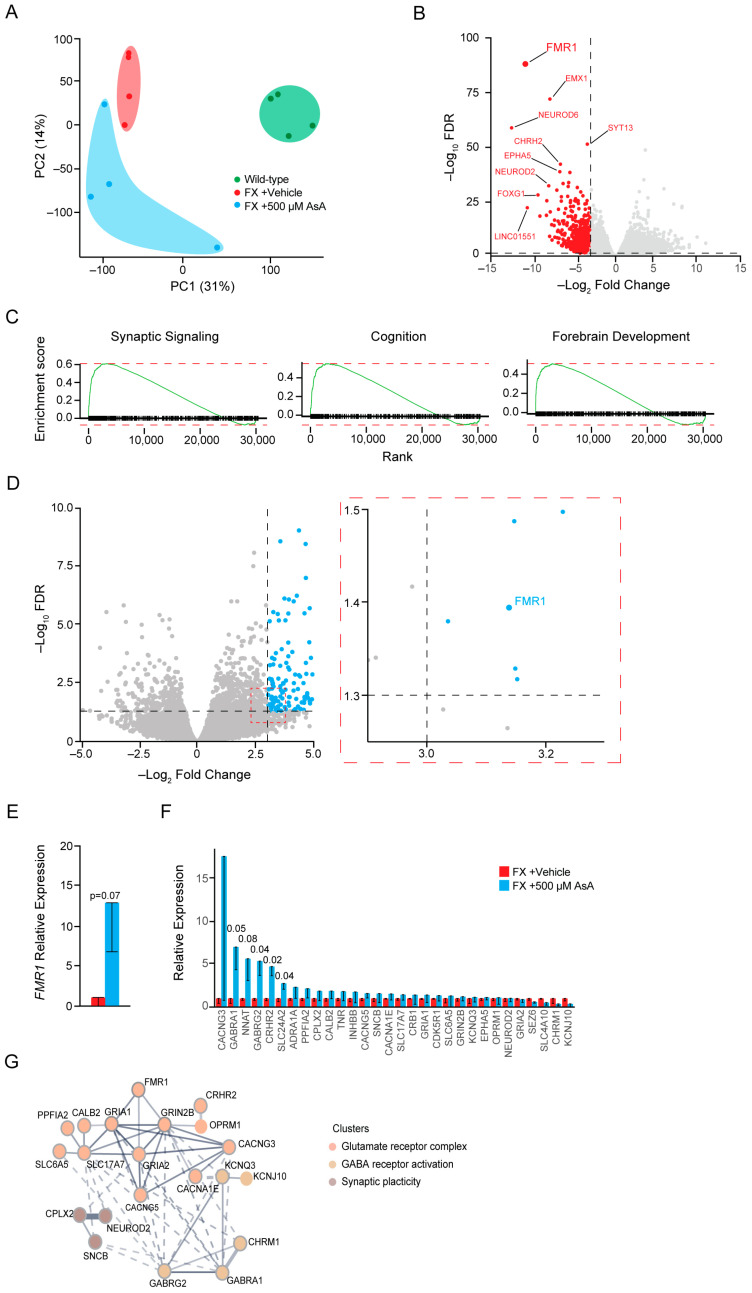
Ascorbic Acid treatment causes changes in gene expression related to neurodevelopment. (**A**) Principal Component Analysis (PCA) of the two most significant principal components (PC1 and PC2) identified by comparing FX vehicle cerebral organoids (n = 4, red), FX + 500 μM AsA cerebral organoids (n = 4, blue), and wild-type cerebral organoids (n = 4, green). (**B**) Volcano plot showing genes that are differentially expressed in FX vehicle cerebral organoids compared to wild-type cerebral organoids. Red indicates genes significantly downregulated in FX cerebral organoids compared with wild-type cerebral organoids. (**C**) Gene enrichment analysis showing families of genes downregulated in FX cerebral organoids compared to wild-type cerebral organoids. (**D**) Volcano plot showing genes that are differentially expressed in FX + 500 μM AsA cerebral organoids compared to FX vehicle cerebral organoids). Blue dots indicate genes with significant upregulation due to AsA treatment. (**E**) Relative expression levels of *FMR1* in FX (red) and FX + 500 μM AsA (blue) cerebral organoids. (**F**) Change in the expression levels of genes found to be most downregulated in FX cerebral organoids compared to wild-type cerebral organoids. Comparison between FX cerebral organoids (red) and FX + 500 μM AsA cerebral organoids (blue). (**G**) String analysis of the most upregulated genes in FX + 500 μM AsA cerebral organoids showing gene families involved in neuronal development, synapse formation, and maturation. All statistical analyses were performed using unpaired one-tail *t*-tests between the FX vehicle control (red) and 500 μM AsA treatment (blue). Error bars are plotted as the ± standard error of the mean.

Principal component analysis (PCA) of the variation across the three groups revealed clear genotype-based separation (Figure 3A). Wild-type cerebral organoids clustered distinctly from both the FX vehicle- and AsA-treated cerebral organoids in the 1st principal component (PC1), indicating that *FMR1* silencing in FX cerebral organoids affected their gene expression profiles. AsA-treated FX cerebral organoids showed greater variability, with looser clustering in both PC1 and PC2, reflecting individual differences in their responses to AsA treatment (Figure 3A). Notably, one AsA-treated cerebral organoid clustered closer to the wild-type organoids in PC1, suggesting that this organoid responded most strongly to AsA and thus had a more robust correction of its transcriptome.

An initial comparison of the cerebral organoid transcriptomes showed that *FMR1* was the most significantly dysregulated gene between FX vehicle-treated and wild-type cerebral organoids, thus supporting this approach (Figure 3B). Most downregulated genes in FX cerebral organoids were involved in neuronal lineage specification and differentiation, including *EMX1*, *NEUROD6*, *EPHA5*, *NEUROD2*, and *FOXG1* (Figure 3B). Enrichment analysis showed that these dysregulated genes were related to neuronal anatomy, function, and development (Supplementary Figure S2A,B). Most pronounced was the downregulation of genes involved in synaptic signaling in cognition and forebrain development (Figure 3C), with 30 of the downregulated genes in FX cerebral organoids involved in synaptic signaling (Supplementary Figure S2A). Gene Set Enrichment Analysis (GSEA) further identified that the most dysregulated genes were related to neuronal development, anatomy, and function (Supplementary Figure S2B).

AsA treatment significantly upregulated *FMR1* by 12.6 ± 0.2-fold compared to vehicle-treated FX cerebral organoids (Figure 3D,E). When analyzing the downregulated genes in the FX cerebral organoids post-AsA treatment, we observed an increased expression of genes involved in neuronal development and synaptic signaling (Figure 3F). Importantly, AsA treatment enhanced the expression of many of the genes involved in neuronal development and synaptic signaling, including *GABRA1*, *NNAT*, *GABRG2*, *CRCH2,* and *SLC24A2* (Figure 3F). STRING analysis of AsA-induced genes identified gene networks related to the ionotropic glutamate receptor complex, GABA receptor activation, and positive regulators of synaptic plasticity (Figure 3G). This supports the notion that AsA can not only promote *FMR1* re-expression but also rescue downstream neuronal aberrations associated with FX. In addition to the correction of downregulated genes in FX cerebral organoids by AsA treatment, AsA treatment partially rescued genes that were significantly upregulated in FX cerebral organoids and brought them closer to wild-type levels; one notable group of genes belonged to the Hox genes (Supplementary Figure S2C).

## 3. Discussion

FX is the most common inherited form of cognitive impairment and mental retardation [1]. Thus, there is a need to find novel therapeutic strategies to treat epigenetic silencing of the *FMR1* gene, which is the root cause of the condition [4,5,9]. The majority of approaches used to model FX and devise therapeutic strategies have focused on animal models [2,35,36]. However, as animals fail to recapitulate the epigenetic silencing of the *FMR1* gene observed in humans due to CGG trinucleotide repeat expansion, therapeutic strategies based on gene knockout models have not been highly effective in the clinic [37]. Therefore, human models using FX iPSCs and their derivatives are promising, as they recapitulate the epigenetic silencing of *FMR1* in FX.

Compound screens in both FX iPSCs and early born neurons showed that the DNMT inhibitor 5AzaC can block methylation of the *FMR1* gene [16,17,18]. Furthermore, deletion of the expanded CGG repeats in *FMR1* can lead to demethylation of the gene [19]. Furthermore, it was shown that de novo R-loops can form in human FX iPSCs, and DNA replication and integrity check systems can excise the CGG repeats, resulting in the re-expression of *FMR1* [38]. These studies exemplified the reversible nature of *FMR1* silencing and the importance of the CGG repeats in the process. This is supported by the finding that targeting demethylases toward the *FMR1* locus in FX iPSCs using dCAS9 can selectively demethylate the locus and reinstate *FMR1* expression [20]. In several studies that attempted to affect the phenotype of FX by pharmacological, genetic, or epigenetic manipulations, the effects were examined at the transcriptome level in adherent neural cells [17,19,20]. We analyzed the effects of AsA on the transcriptome of organotypic cerebral organoids.

These studies provide a foundation for possible approaches to treat FX in humans. However, 5AzaC can be cytotoxic and may have consequences in children developing FX. In addition, it will be technically challenging to target all cells of the brain with putative genetic approaches to edit out CGG repeats of FX *FMR1*, for example, by using CRISPR technology or R-loops for de novo editing or guided demethylases targeting the *FMR1* locus. Hence, a pharmacological approach could help circumvent these problems.

Our human FX patient-derived iPSCs and cerebral organoid models recapitulated the epigenetic silencing of *FMR1*. We show that both FX iPSCs and cerebral organoids responded to AsA treatment, with reduced methylation of the *FMR1* locus and increased levels of *FMR1* transcripts. In addition, AsA treatment results in a degree of rescue of organoid morphology in FX organoids and an increase in defective neuronal gene expression. Although demethylation of the CpG island upstream of the *FMR1* gene in FX iPSCs as a population was uniform and homogenous in response to AsA treatment, the responses of individual cerebral organoids were variable. We observed that 38–45% of the organoids analyzed showed a positive response in organoid morphology, CpG island demethylation, and induction of *FMR1* gene expression.

These findings show that AsA treatment has a pronounced effect on FX neural tissue; however, only a fraction of the cells seem to respond in these assays. Why FX organoids show heterogeneity in response to AsA remains to be determined. One reason could be that cerebral organoids are not uniform, and some are much larger than others. Technically, organoid size and the ensuing differences in the efficacy of AsA penetration may play a role in the response of cells within organoids. Additionally, cerebral organoids are heterogeneous in their differentiation stage and cellular composition, which could also lead to differences in their biological responses. These limiting factors of the cerebral organoid systems must be considered in future studies on neuronal differentiation of FX iPSCs. In the future, single-cell analyses at the genomic, RNA, and protein levels may be able to address differences in cell type-specific responses to AsA.

Our findings demonstrate that AsA treatment can counteract, to some degree, FX-induced neurodevelopment gene expression aberrations, particularly neuronal differentiation and synapse formation. It is conceivable that a reduction in the expression of these genes in FX contributes to the developmental and cognitive abnormalities observed in patients with FX. It is also plausible that AsA modification of *FMR1* methylation and expression could have a positive effect on neurodevelopment in patients with FX. However, we cannot exclude the possibility that the changes induced by AsA are not restricted to the activation of the *FMR1* gene and may have more global effects on gene expression. In the future, genome-wide restricted bisulfite sequencing should be performed to assess changes in the methylation status of other loci.

Two previous studies used FX iPSCs and differentiated them into cerebral organoids to study and characterize the fundamental processes of human FX. One study generated *FMR1* knockout iPSCs by truncating the *FMR1* gene using CRISPR/Cas9 gene editing. Although useful for examining the role of FMRP in human neural cells in vitro, this model system does not utilize the epigenetic silencing that causes FX [39]. Another study found that molecular defects in FX cerebral organoids could be rescued by inhibiting the phosphoinositide 3-kinase pathway [40]. How the phosphoinositide 3-kinase pathway is potentially involved in FX remains unclear, and whether the methylation status of *FMR1* was altered was not addressed [40]. In summary, our study showed for the first time that AsA can target the root cause of FX, the epigenetic silencing of *FMR1,* in both FX iPSCs and cerebral organoids. To our knowledge, our study is the first time that a human cerebral organoid model has been used to demonstrate a reversal of epigenetic silencing of the endogenous *FMR1* gene without genetic intervention.

## 4. Materials and Methods

### 4.1. iPSC Cell Culture

FX52 (FX) and IPSO (wild-type) cell lines were used in these experiments [15]. FX52 and IPSO were cultured and maintained as described in previous studies [15]. Plating of cells for testing Ascorbic Acid concentration was determined by plating 100,000 cells per well in a 6-well plate. Cultures were maintained for 6 days until they were collected for harvesting DNA and RNA. FX52 and IPSO iPSCs were cultured using mTeSR1 media (Stem Cell Technologies, Vancouver, BC, Canada, mTeSR1 Basal Medium, Cat#05850 and mTeSR1 5x supplement, Cat#05851) on Matrigel (BD Bioscience, Franklin Lakes, NJ, USA, hESC qualified Matrigel, Cat#354277) at 37 °C, 5% CO_2_ with daily media change. Cells were passaged enzymatically every 6 days using StemPro Accutase (Gibco, Life Technology, Carlsbad, CA, USA, Cat#A1110501). Cells were grown in mTeSR1 medium in the presence of 10 μM Y-27632 ROCK inhibitor (Miltenyi Biotec, Bergisch Gladbach, Germany, Cat #130-104-169) after passage.

iPSCs vehicle-controls contained mTeSR1 supplemented with an additional 15 mM HEPES Buffer (Sigma, Burlington, MA, USA, HEPES 1 M, Cat#H0887). Ascorbic Acid (Sigma, L-Ascorbic Acid 100G, Cat#A92902-100G) was solubilized in ddH_2_O and supplemented at the appropriate concentration to mTeSR1 plus 15 mM HEPES supplementation.

### 4.2. Cerebral Organoid Differentiation

Cerebral organoid differentiation was performed exactly according to Lancaster et al. [33]. The only deviation was with every media change; organoid culture media was supplemented with 15 mM HEPES (vehicle control) (Sigma, HEPES 1 M, Cat#H0887)and 500 μM Ascorbic Acid (Sigma, L-Ascorbic Acid 100 G, Cat#A92902-100G). Organoids were harvested at day 50 for analysis.

### 4.3. DNA and RNA Extraction

DNA extraction for cerebral organoids was performed using QIAamp DNA Mini Kit (50) (Qiagen, Hilden, Germany, QIAamp DNA Mini Kit (50), Cat#51304) following instructions exactly as described in the protocol. DNA was stored at −20 °C.

RNA extraction from cerebral organoids was performed using Trizol extraction. Organoids were lysed directly in Trizol (Invitrogen, Waltham, MA, USA, Trizol, Cat #15596018). RNA was isolated according to the manufacturer’s instructions. Cells were directly sorted into 1mL of Trizol. RNA was extracted from the aqueous phase after the addition of 200 μL of chloroform, precipitated with an equal volume of isopropanol, and washed once in fresh 80% ethanol. The RNA pellet was re-suspended in 20 μL ddH_2_O at 50 °C for 10 min and then kept on ice. RNA was stored at −20 °C.

Simultaneous DNA and RNA extraction from iPSCs was performed using the AllPrep DNA/RNA Micro Kit (50) (Qiagen, AllPrep DNA/RNA Micro Kit (50), Cat#80284) and performed exactly according to the manufacturer’s instructions. DNA and RNA were stored at −20 °C.

### 4.4. RT-PCR

RT-PCR to characterize gene expression levels of *FMR1* was performed using TaqMan Assay primers (*FMR1*: Hs00924547_m1, ThermoFisher, Waltham, MA, USA) and TaqMan Fast Advanced Master Mix for qPCR (ThermoFisher, REF#4444557). *FMR1* expression levels were normalized to *GAPDH* (*GAPDH*: Hs02786624_g1, ThermoFisher).

### 4.5. Pyrosequencing

Genomic DNA was extracted using a QIAamp DNA Mini Kit (Qiagen, Hilden, Germany), including RNase A treatment, according to the manufacturer’s protocol. Two micrograms of genomic DNA from each sample was bisulfite-treated using the EZ DNA Methylation Kit (Zymo Research, Orange, CA, USA), following the manufacturer’s instructions, and stored at −80 °C until further use.

Pyrosequencing was used to quantify DNA methylation levels in the CpG island at the *FMR1* transcription start site. Primers were designed to amplify a 190 bp sequence containing 22 CpGs located 150 bp upstream of the CGG repeat sequence. PCR was performed in two steps. The first PCR used a forward primer (5′-GTTATTGAGTGTATTTTTGTAGAAATG-3′) and a reverse primer with an 11-base tag sequence (5′-GCCCCCGCCCGCCCTCTCTCTTCAAATAACCTAAAAAC-3′). The second PCR used a nested forward primer (5′-GAGTGTATTTTTGTAGAAATGGG-3′) and a universal reverse primer with the same 11-base tag sequence (5′-(biotin)GCCCCCGCCCG-3′), which was biotin-labeled at the 5′ end and HPLC-purified (Microsynth AG, Balgach, Switzerland). The first PCR was performed in a 25 μL reaction volume using FastStart Taq DNA polymerase (Roche Diagnostics, Indianapolis, IN, USA). After initial denaturation at 95 °C for 6 min, the cycling conditions consisted of 35 cycles of denaturation at 95 °C for 30 s, annealing at 57 °C for 30 s, and elongation at 72 °C for 45 s. The amplified fragment was diluted 1000-fold and used as a template for the second PCR. The second PCR was carried out in a 50 μL reaction volume, with initial denaturation at 95 °C for 5 min, followed by 25 cycles of denaturation at 95 °C for 30 s, annealing at 52 °C for 30 s, and elongation at 72 °C for 45 s. A 10 μL aliquot of the PCR product was directly used for pyrosequencing with the PyroMark Q24 Pyrosequencing system, following the manufacturer’s instructions (Qiagen). For each sample, two sequencing runs were performed using either the nested forward primer as the sequencing primer or an internal sequencing primer S1 (5′-GTTTTTTATTAAGTT-3′) to assess the methylation status of all 22 CpGs. The ratio of cytosine to thymine at each analyzed CpG site, quantified by assessing the peak height, was exported using PyroMark Q24 Advanced Application Software Version 3.0.0. The percentage represents the average methylation level of all sequenced PCR products for the respective CpG site analyzed within the assay.

### 4.6. RNASeq and Bioinformatic Analysis

All the graphs for Figure 3 and Supplementary Figure S2 were made using RStudio 4.4.1. The data were prepared by collecting the RNASeq analysis, aligning it to human (GRCh38 human; https://www.gencodegenes.org/human/ (accessed on 30 June 2024)) and mouse (GRCm39 mouse; https://www.gencodegenes.org/mouse (accessed on 30 June 2024)) genomes (STAR) and then to eliminate the mouse aligned reads (XenofilteR), and finally we conducted genome-wide expression analysis and normalization by TTM. Then, the values table was taken and processed to build in the figures. All RNA-seq files are deposited in ArrayExpress under accession number E-MTAB-14543.

### 4.7. Statistical Analysis

All statistical analyses for Figure 1 and Figure 2 were performed using the statistics functions of GraphPad Prism Version 9.2.0 (283). All tests were performed as one-tailed *t*-tests comparing FX versus FX Ascorbic Acid treated. Error bars are all plotted as ±Standard Error of the Mean (SEM). For statistical significance in figures, standard convention follows: * *p* < 0.05; ** *p* < 0.01; *** *p* < 0.001; **** *p* < 0.0001.

## Data Availability

SRA for all RNASeq samples will be deposited soon, and access codes will be provided to the reviewers.

## Supplementary Materials

The following supporting information can be downloaded at https://www.mdpi.com/article/10.3390/ijms252312718/s1.
