# Supplementary material for: Ascorbic Acid Ameliorates Molecular and Developmental Defects in Human-Induced Pluripotent Stem Cell and Cerebral Organoid Models of Fragile X Syndrome
Source: Int J Mol Sci. 2024 Nov 26;25(23):12718. doi: 10.3390/ijms252312718 (PMC11641479; doi:10.3390/ijms252312718)
Supplement: Supplementary file 1 [file ijms-25-12718-s001.zip › Supplementary Figure 1 Legend copy.pdf]

Supplementary Figure S1. (A). Top row, representative images of cerebral organoids with proper morphology by day 50; scale bar for left, center and right panel are 2500 μm, 4000 μm and 4000 μm respectively. Bottom row, representative images of cerebral organoids with improper morphology or lacking dense compact tissue and containing cystic structures; scale bar for left, center and right panel are 2500 μm, 2000 μm and 2500 μm respectively. (B). Plots showing individual FX vehicle control and wild-type cerebral organoid methylation status across the 14 CpGs. Dashed red lines indicate FX vehicle controls, thick red line is the average of all FX vehicle controls. Dashed green lines indicate wild-type cerebral organoids, thick green line is the average of all wild-type cerebral organoids. (C). Plots showing individual FX +AsA and wild-type cerebral organoid methylation status across the 14 CpGs. Dashed grey lines indicate FX +AsA non-responder organoids, thick grey line is the average of all FX +AsA non-responder cerebral organoids. Dashed blue lines indicate FX +AsA responder cerebral organoids, thick blue line is the average of all FX +AsA responder cerebral organoids. Dashed green lines indicate wild-type cerebral organoids, thick green line is the average of all wild-type cerebral organoids.
