# Supplementary material for: Ascorbic Acid Ameliorates Molecular and Developmental Defects in Human-Induced Pluripotent Stem Cell and Cerebral Organoid Models of Fragile X Syndrome
Source: Int J Mol Sci. 2024 Nov 26;25(23):12718. doi: 10.3390/ijms252312718 (PMC11641479; doi:10.3390/ijms252312718)
Supplement: Supplementary file 1 [file ijms-25-12718-s001.zip › Supplementary Figure 2 legend copy.pdf]

Supplementary Figure S2. (A). Expression levels of most downregulated genes in FX cerebral organoids (red) and basal expression levels of the same genes in wild-type cerebral organoids (green). All statistics performed were unpaired one-tail t-test between FX vehicle control (red) and wild-type cerebral organoids (green); * $p < 0.05$; ** $p < 0.01$; *** $p < 0.001$. (B). Genes families most significantly downregulated in FX cerebral organoids compared to wild-type cerebral organoids. Error bars plotted are ± standard error of the mean. (C) Relative expression of upregulated gene in FXS cells that are partially corrected by the treatment of AsA. All statistics performed were unpaired one-tail t-test between FX vehicle control (red) and FX + AsA organoids (blue); * $p< 0.05$. Error bars plotted are ± standard error of the mean.
