# Supplementary figures and images for: Ascorbic Acid Ameliorates Molecular and Developmental Defects in Human-Induced Pluripotent Stem Cell and Cerebral Organoid Models of Fragile X Syndrome
Source: Int J Mol Sci. 2024 Nov 26;25(23):12718. doi: 10.3390/ijms252312718 (PMC11641479; doi:10.3390/ijms252312718)

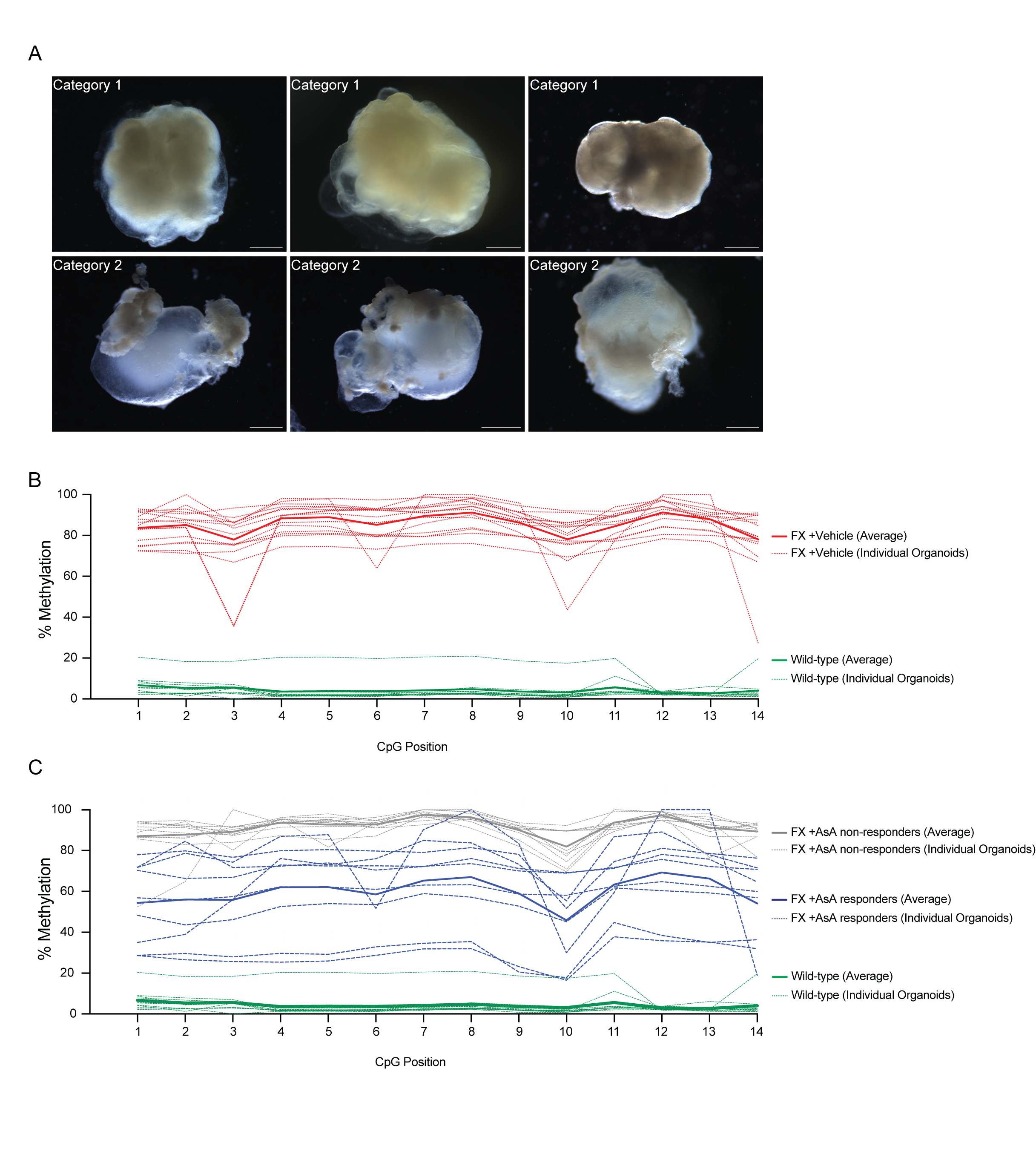

Supplement: Supplementary file 1 [file ijms-25-12718-s001.zip › Supplementary Figure 1 for Figure 2 copy.tif]

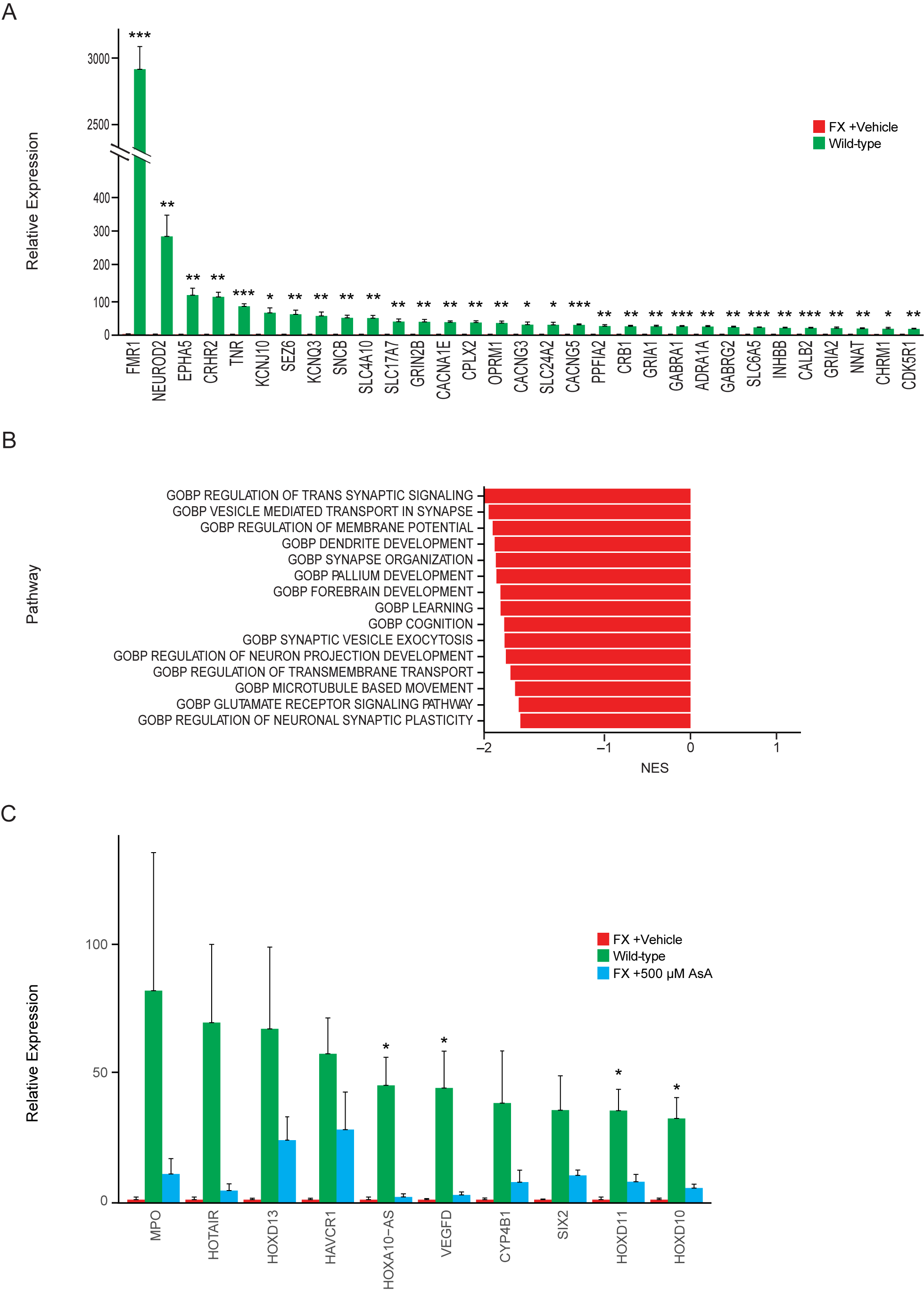

Supplement: Supplementary file 1 [file ijms-25-12718-s001.zip › Supplementary Figure 2 for Figure 3 copy.tif]
